# Wall teichoic acid-dependent phagocytosis of intact cell walls of *Lactiplantibacillus plantarum* elicits IL-12 secretion from macrophages

**DOI:** 10.3389/fmicb.2022.986396

**Published:** 2022-08-09

**Authors:** Naoya Kojima, Shohei Kojima, Shin Hosokawa, Yoshiki Oda, Daisuke Zenke, Yuta Toura, Emi Onohara, Shin-ichi Yokota, Masato Nagaoka, Yasuhiro Kuroda

**Affiliations:** ^1^Department of Applied Biochemistry, Tokai University, Hiratsuka, Japan; ^2^Technology Joint Management Office, Tokai University, Hiratsuka, Japan; ^3^Department of Microbiology, Sapporo Medical University School of Medicine, Sapporo, Japan; ^4^Yakult Central Institute, Kunitachi-shi, Japan

**Keywords:** actin remodeling, cell wall teichoic acid, IL-12, intact cell wall, *Lactiplantibacillus plantarum*, macrophage, phogocytosis

## Abstract

Selected lactic acid bacteria can stimulate macrophages and dendritic cells to secrete IL-12, which plays a key role in activating innate and cellular immunity. In this study, we investigated the roles of cell wall teichoic acids (WTAs) displayed on whole intact cell walls (ICWs) of *Lactiplantibacillus plantarum* in activation of mouse macrophages. ICWs were prepared from whole bacterial cells of several lactobacilli without physical disruption, and thus retaining the overall shapes of the bacteria. WTA-displaying ICWs of several *L. plantarum* strains, but not WTA-lacking ICWs of strains of other lactobacilli, elicited IL-12 secretion from mouse bone marrow-derived macrophages (BMMs) and mouse macrophage-like J774.1 cells. The ability of the ICWs of *L. plantarum* to induce IL-12 secretion was abolished by selective chemical elimination of WTAs from ICWs, but was preserved by selective removal of cell wall glycopolymers other than WTAs. BMMs prepared from TLR2- or TLR4-deficient mouse could secret IL-12 upon stimulation with ICWs of *L. plantarum* and a MyD88 dimerization inhibitor did not affect ICW-mediated IL-12 secretion. WTA-displaying ICWs, but not WTA-lacking ICWs, were ingested in the cells within 30 min. Treatment with inhibitors of actin polymerization abolished IL-12 secretion in response to ICW stimulation and diminished ingestion of ICWs. When overall shapes of ICWs of *L. plantarum* were physically disrupted, the disrupted ICWs (DCWs) failed to induce IL-12 secretion. However, DCWs and soluble WTAs inhibited ICW-mediated IL-12 secretion from macrophages. Taken together, these results show that WTA-displaying ICWs of *L. plantarum* can elicit IL-12 production from macrophages *via* actin-dependent phagocytosis but TLR2 signaling axis independent pathway. WTAs displayed on ICWs are key molecules in the elicitation of IL-12 secretion, and the sizes and shapes of the ICWs have an impact on actin remodeling and subsequent IL-12 production.

## Introduction

Cell walls of Gram-positive bacteria consist of thick and multiple peptidoglycan layers and glycopolymers attached to the peptidoglycans. The cell wall glycopolymer components of Gram-positive bacteria are classified into three groups according to their structural characterization: (i) teichoic acids, which are phosphodiester-linked polymers of alditol (glycerol or ribitol) phosphates, glycosylalditol phosphates, and sugar phosphates; (ii) teichuronic acids, which are acidic polysaccharides containing uronic acids; and (iii) other neutral and acidic polysaccharides. These glycopolymers are highly diverse and species- and strain-specific (Weidenmaier and Peschel, 2007). Among them, teichoic acids are the most abundant cell wall glycopolymer in many Gram-positive bacteria and comprise up to 60% of dry cell wall weight; hence, these molecules are believed to have important physiological roles (Brown et al., 2013).

There are two distinct types of teichoic acids in Gram-positive bacteria: peptidoglycan-attached teichoic acids referred to as wall teichoic acids (WTAs); and lipid-linked teichoic acids anchored into the plasma membrane, which are referred to as lipoteichoic acids (LTAs) (Morath et al., 2005; Brown et al., 2013; Shiraishi et al., 2016). LTAs are recognized as strong immunomodulatory components in cell walls of Gram-positive bacteria (Ginsburg, 2002; Draing et al., 2008; Hong et al., 2014). For instance, LTAs purified from some strains of lactobacilli can induce a potent pro-inflammatory response in immune cells *in vitro* by interaction with TLR2 on host cells (Matsuguchi et al., 2003; Hirose et al., 2010). In contrast, immunomodulation by WTAs is not well understood, although WTAs have numerous roles in cell walls, including regulation of cell division and autolytic activity, regulation of ion homeostasis, and involvement in bacterial attachment and colonization (Brown et al., 2013).

Lactobacilli are common members of the indigenous microbiota and are used in many foods as probiotics, which have been shown to have a wide range of biological activities including host immune modulation (Kemgang et al., 2014; Sánchez et al., 2017). Thus lactobacilli are believed to have roles in development and maintenance of the immune system. Indeed, whole bacterial cells of some probiotic lactobacilli can activate innate and adaptive immune responses, accompanied by significant production of inflammatory cytokines such as IL-12, a key cytokine in activating innate immunity and subsequent cellular immunity (Hessle et al., 1999; Morita et al., 2002; Hisbergues et al., 2007; Rigaux et al., 2009).

We have shown that oligomannose-coated liposomes (OMLs) with a diameter of 1 μm can elicit antigen-specific Th1 immune responses in mice. This elicitation of Th1 immunity relies on carbohydrate-dependent phagocytosis of OMLs by mononuclear phagocytes and subsequent induction of IL-12 production by the cells. Particle size of OMLs has an impact on IL-12 production from the cell; OMLs of diameter ≥600 nm are required to induce IL-12 production (Matsuoka et al., 2021). Phagocytosis is a cellular process to ingest and eliminate particles over 500 nm in diameter such as microorganisms and hence recognized as fundamental process in immunity (Uribe-Querol and Rosales, 2020). Therefore, we hypothesize that phagocytosis of large carbohydrate-decorated particles by mononuclear phagocytes appears to be important for cytokine production and elicitation of innate and cellular immunity.

The whole intact cell walls (ICWs), which are prepared from the whole bacterial cells without physical disruption by sequential treatment with sodium dodecyl sulfate (SDS), pronase, and nuclease, and hence, do not contain proteins, nucleic acids, and lipid components including LTAs, have been used for investigation of roles of peptidoglycan components of Gram-positive bacteria in immune responses (Sekine et al., 1985; Shida et al., 2006; Kaji et al., 2010). ICWs morphologically retain the bacterial shape and preserve an ICW (peptidoglycan) structure including peptidoglycan-attached glycopolymers such as WTAs; hence, ICWs can be thought of as a kind of large carbohydrate-decorated particles. In fact, ICWs prepared from some lactobacilli can induce IL-12 production from peritoneal exudative macrophages (Shida et al., 2006), just like OMLs. Of note, the whole peptidoglycan without cell wall glycopolymers (hydrogen fluoride-treated ICWs) failed to induce cytokine production, indicating that the glycopolymers attached to ICWs are somehow related to cytokine production from cells stimulated by ICWs of lactobacilli. As soluble cell wall glycopolymer-peptidoglycan complexes cannot induce cytokine production (Shida et al., 2006), glycopolymers may have immunomodulatory activities when displayed on large particles such as ICWs and liposomes. However, it is unclear why ICWs but not glycopolymer-peptidoglycan complexes have immunomodulatory activities and whether the glycopolymers on ICWs are functionally involved in the immunomodulatory activities.

As strains of *Lactiplantibacillus plantarum* (recently renamed from *Lactobacillus plantarum*) express WTAs (Tomita et al., 2012) and exhibit immunomodulatory activities, including cytokine induction (Hisbergues et al., 2007; Rigaux et al., 2009; Garacia-Gonzales et al., 2021), we assumed that WTAs expressed on *L. plantarum* might be involved in these activities. To clarify immunomodulatory activities of cell wall glycopolymer attached to peptidoglycan and requirement of carbohydrate-dependent phagocytosis of large carbohydrate-decorated particles for elicitation of immune responses, in this study, we examined the responses of murine macrophages by stimulation with ICWs of *L. plantarum* as large carbohydrate-decorated particles. For this purpose, we prepared various types of chemically modified ICWs based on the chemical structure of cell wall glycopolymers including WTA (Kojima et al., 1985a,b). We found that WTA-displaying ICWs of *L. plantarum* can elicit IL-12 production from macrophages *via* actin-dependent phagocytosis but TLR2 signaling axis independent pathway, and concluded WTAs displayed on ICWs play a key molecule in elicitation of ICW-mediated IL-12 secretion.

## Materials and methods

### Bacterial strains

The following bacterial strains were used in the study: *Lactobacillus gasseri* JCM 1131*^T^* and JCM 5814; *Limosilactobacillus fermentum* (previously *Lactobacillus fermentum*) ATCC 9338; *Lactobacillus helveticus* YIT 0049; *L. plantarum* TUA 5099L, NRIC 1067*^T^*, and YIT 0139; and *Lacticaseibacillus rhamnosus* (previously *Lactobacillus rhamnosus*) ATCC 7469*^T^*. These bacteria were initially obtained from the American Type Culture Collection (ATCC), the Japan Collection of Microorganisms (JCM), the Nodai Research Institute Culture Collection (NRIC), Tokyo University of Agriculture (TUA), and Yakult Central Institute (YIT). All strains were cultured at 37°C for 20 h in lactobacilli-MRS broth (Difco), washed with 20 mM Tris–HCl (pH 7.4), and then heated at 100°C for 20 min to obtain heat-killed bacteria.

### Chemicals and reagents

Chemicals and cell culture reagents were obtained from FUJIFILM Wako unless otherwise stated. Pronase from *Streptomyces griseus* and murine macrophage colony stimulating factor (M-CSF) were purchased from Roche Diagnostics and PeproTech, respectively. Benzonase^®^ nuclease, chlorpromazine, IκB kinase (IKK) inhibitor II, interleukin-1 receptor associated kinase (IRAK) 1/4 inhibitor I, latrunculin B, lipopolysaccharide (LPS) from *Escherichia coli* 0111:B4 were from Sigma-Aldrich. Pam_3_CSK_4_, a synthetic TLR2 ligand, were from Invivogen. A MyD88 dimerization inhibitor, ST2825, was obtained from ChemScene LCC. Diethylaminoethyl (DEAE)-cellulose (DE-52) and DEAE-Sepharose^®^ were form Whatman and Sigma-Aldrich, respectively. *N*-hydroxy-succinimidyl (NHS)-fluorescein and a PerCP-Cy5.5-labeled anti-mouse CD11b monoclonal antibody (clone M1/70) were obtained from Thermo Fisher Scientific and BD Pharmingen, respectively.

### Preparation of intact cell walls

Intact cell walls of each strain were prepared from heat-killed bacteria, as described previously (Sekine et al., 1985; Shida et al., 2006) with small modifications. Briefly, heat-killed bacteria were boiled in 1% SDS for 15 min and washed with distilled water three times and acetone once to remove SDS. The resulting precipitates were then digested with 1 mg/ml of pronase in 50 mM Tris–HCl (pH 7.4) at 37°C for 20 h. After extensive washing with distilled water, the precipitates were digested with 10 U/ml of Benzonase nuclease in 50 mM Tris–HCl (pH 7.4) at 37°C for 20 h, treated again with 1% SDS, and then stirred in chloroform/methanol/water (10:10:3) overnight at room temperature to remove lipids. The delipidated material was treated sequentially with nuclease and pronase in 50 mM Tris–HCl (pH 7.4). Insoluble material obtained by centrifugation at 8,000 × *g* for 15 min was exhaustively washed with distilled water, lyophilized, and used as ICWs. Scanning electron micrographs of ICWs were obtained using a JEOL JSM-1610LV instrument.

### Preparation of bone marrow-derived macrophages and cell culture

Animal experiments were conducted in compliance with the ethical requirements of the Animal Committee at Tokai University (approval numbers 191002, 201041, and 212008). For preparation of bone marrow-derived macrophages (BMMs), 6–8-week-old female C57BL/6 mice were purchased from Shizuoka Laboratory Animal Corp, and TLR2-deficient mice and TLR4-deficient mice on a C57BL/6 background were purchased from Oriental BioService, Inc. The mice were housed in a specific pathogen-free environment under standard housing conditions. BMMs were prepared using the protocol described by Zang et al. (2008) with minor modifications. Briefly, cells were obtained from bone marrow and cultured (5 × 10^5^ cells/ml) with 10 ml of RPMI1640 medium supplemented with 10% FBS, 100 U/ml penicillin, 100 μg/ml streptomycin and 50 μM 2-mercaptoethanol (complete medium) containing 20 ng/ml M-CSF in a 10-cm petri dish. On day 3, another 5 ml of complete medium containing M-CSF was added to the dish. On day 7, adherent cells were detached by Cellstripper non-enzymatic cell dissociation solution (Corning). The resulting cells, over 90% of which expressed CD11b, were used as BMMs. The murine macrophage-like cell line J774.1 was obtained from the Japanese Collection of Research Bioresources. The cells were cultured with complete medium.

### Stimulation of macrophages with intact cell walls

J774.1 cells (5 × 10^5^/ml) or BMMs (5 × 10^4^/ml) suspended in complete medium were placed in a 1.5-ml tube (600 μL) and ICWs of each strain were added at a final concentration of 10 μg/ml. The cells were incubated at 37°C for 30 min with gentle rotation, and then seeded in a well of a 24-well culture plate and cultured for 24 h. As a positive control, cells were stimulated with 500 ng/ml of LPS or 1 μg/ml of Pam_3_CSK_4_ (Invivogen). After 24 h, culture supernatants were collected and IL-12p40 in the supernatants was quantified using a commercial ELISA kit (BD). For inhibition of phagocytosis, cells were first treated with cytochalasin D (1 μM), latrunculin B (1 μM), jasplakinolide (0.1 μM) or chlorpromazine (1 μg/ml) in complete RPMI1640 containing 0.1% DMSO for 30 min. For inhibition of TLR signaling, cells were first treated with ST2825 (10 μM), IKK inhibitor II (10 μM), or IRAK 1/4 inhibitor I (0.5 μM) in complete RPMI1640 containing 0.1% DMSO for 30 min. After 30 min incubation with respective inhibitors, the cells were stimulated with ICWs or LPS, respectively.

### Treatment of intact cell walls with mild acid and mild base

Intact cell walls obtained from each strain were subjected to mild acid or mild base treatment, as described previously (Kaya et al., 1984; Kojima et al., 1985a,b). For *N*-acetylation, ICWs (100 mg) were treated with 1 ml of acetic anhydride in 20 ml of saturated sodium carbonate solution for 20 h at 4°C. After extensive washing with distilled water, the resulting *N*-acetylated ICWs (*N*Ac-ICWs) were treated with 0.1 M glycine-HCl (pH 2.5) at 100°C for 30 min at a concentration of 5 mg/ml. After centrifugation at 8,000 × *g* for 15 min, the precipitates were used as acid-treated *N*Ac-ICWs after washing with distilled water. The supernatants were dialyzed against distilled water, lyophilized, and used for analysis of cell wall glycopolymers. Alternatively, unmodified ICWs (100 mg) were directly treated with 0.1 M glycine-HCl (pH 2.5) at 100°C for 30 min or with 0.5 M NaOH at 37°C for 30 min, and the resulting insoluble materials were used as acid-treated ICWs (AICWs) and base-treated ICWs (BICWs), respectively. AICWs or BICWs were then treated with 0.1 M glycine-HCl (pH 2.5) at 100°C for 30 min after *N*-acetylation. After centrifugation at 8,000 × *g* for 15 min, precipitates were used as acid-treated *N*Ac-AICWs and acid-treated *N*Ac-BICWs (Figure 4A). Supernatants obtained by mild acid treatment were dialyzed against distilled water, lyophilized, and used for analysis of cell wall glycopolymers. Hydrogen fluoride (HF)-treated ICWs were prepared by treatment of ICWs with 47% HF for 16 h at 4°C. This treatment cleaves all the phosphodiester bonds in ICWs (Sieling et al., 1992), and thus, cell wall glycopolymers are completely removed from ICWs.

**FIGURE 1:**
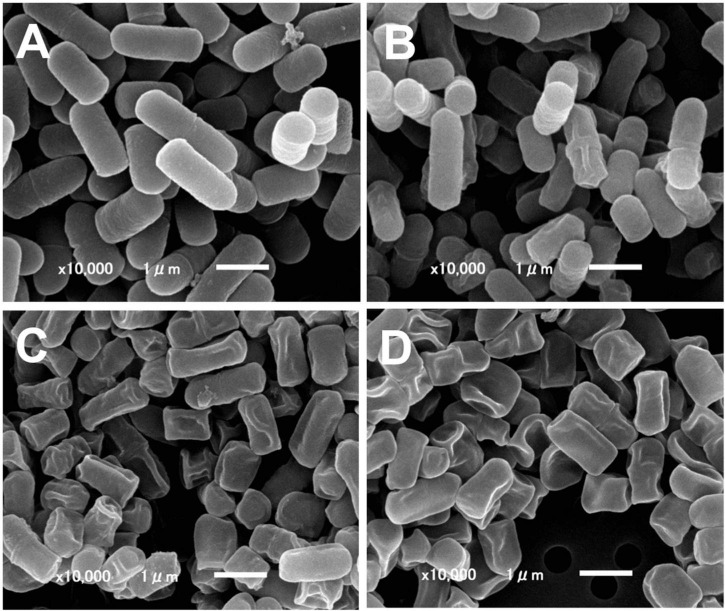
Scanning electron microscopy of ICWs of *L. plantarum* TUA 5099L. **(A)** Heat-killed cells. **(B)** Intact cell walls. **(C)** Acid-treated ICWs. **(D)** Base-treated ICWs.

**FIGURE 2:**
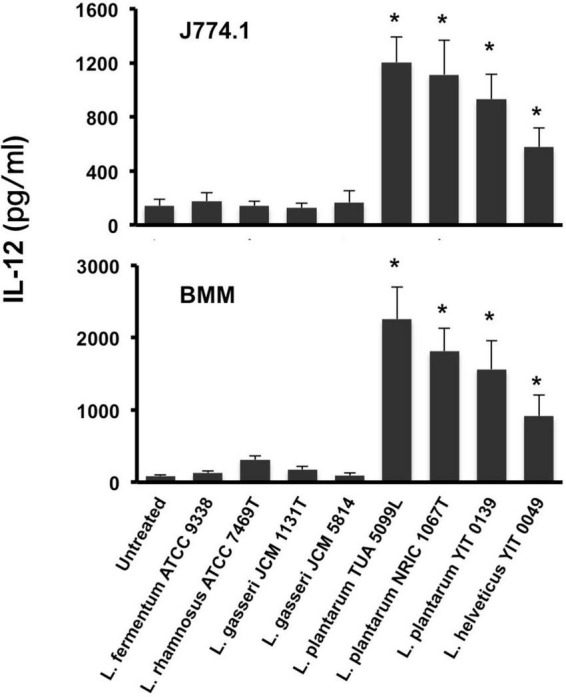
IL-12 secretion from macrophages stimulated with ICWs of lactobacilli. ICWs were prepared from heat-killed cells of several lactobacilli by SDS, pronase, and nuclease treatments. J774.1 cells (5 × 10^5^/ml, upper panel) and BMMs (5 × 10^4^/ml, lower panel) were cultured with 10 μg/ml of ICWs for 24 h and IL-12 secreted in the culture supernatant was determined. Each bar is the mean ± SE for five independent experiments. The results were reproducible for at least three independent ICW preparations for each strain. *P < 0.01 vs. untreated cells.

**FIGURE 3:**
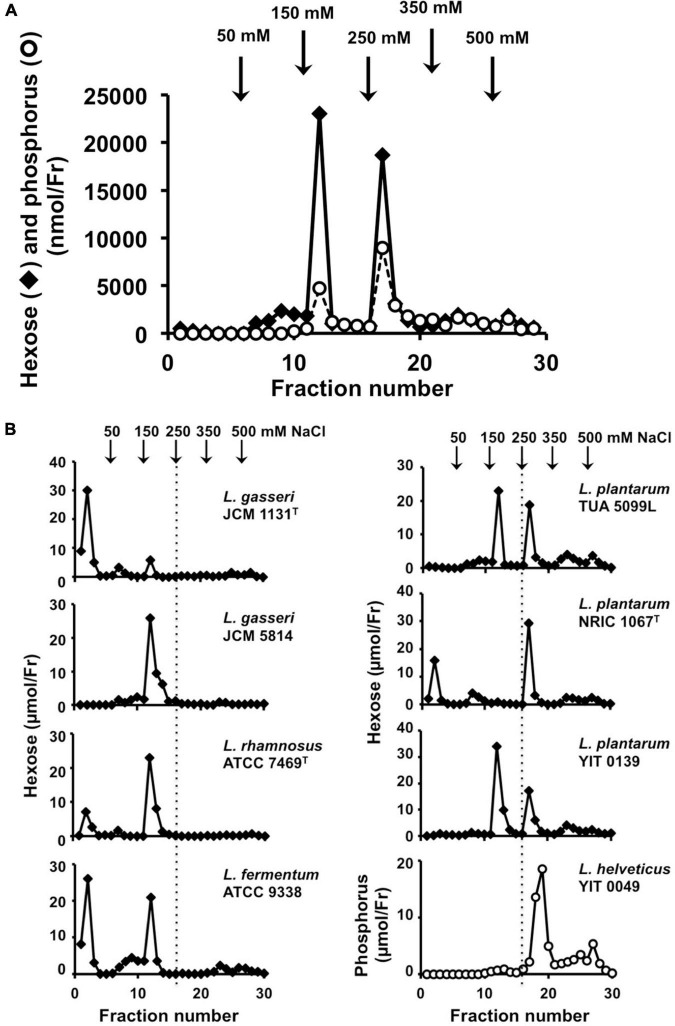
Analysis of cell wall glycopolymers isolated from *N*-acetylated ICWs of lactobacilli. **(A)** ICWs of *L. plantarum* TUA 5099L were *N*-acetylated and treated with 0.1 M glycine-HCl, pH 2.5 at 100°C for 30 min. After centrifugation at 8,000 × *g* for 15 min, supernatants were recovered, dialyzed against 5 mM Tris–HCl (pH 7.4), and applied to a DEAE-cellulose column. The column was eluted in a stepwise manner with 5 mM Tris–HCl (pH 7.4) containing 50, 150, 250, 350, and 500 mM NaCl. Fractions (5 ml) were collected and hexoses and phosphorus in each fraction were determined. **(B)** Chromatography of glycopolymers isolated from *N*-acetylated ICWs of eight strains of lactobacilli. Glycopolymers isolated from *N*-acetylated ICWs of each strain were subjected to chromatography on a DEAE-cellulose column, as described above, and hexose or phosphorus was determined. The figure shows representative chromatograms of at least three independent ICW preparations of each strain. Other ICW preparations also gave the same chromatograms. Note that *L. plantarum* TUA 5099L, NRIC 1067*^T^*, and YIT 0139 and *L. helveticus* YIT 0049 contain glycopolymers eluted at 250 mM NaCl.

**FIGURE 4:**
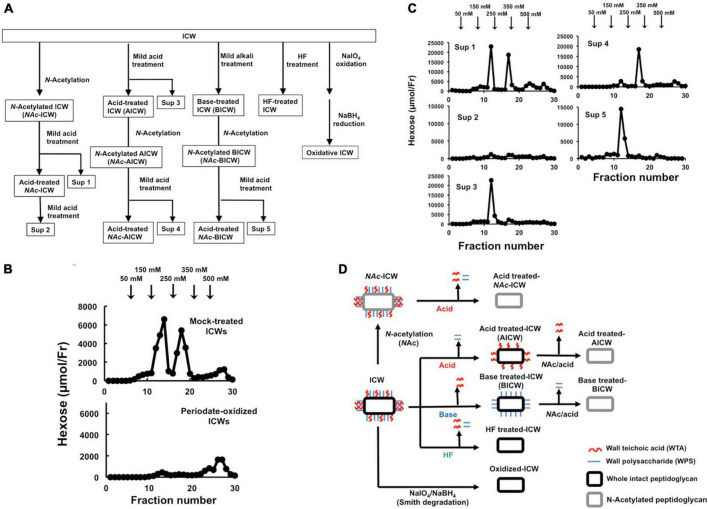
Chemical modification of ICWs of *L. plantarum* TUA 5099L. **(A)** Flowchart of treatments of ICWs with mild acid (0.1 M glycine-HCl, pH 2.5, 100°C, 30 min), mild base (0.5 M NaOH, 37°C, 30 min), aqueous hydrogen fluoride (47% HF, 4°C, 16 h), and periodate (0.1 M NaIO_4_, pH 4.5, 4°C 48 h). **(B)** Smith degradation of ICWs. Periodate-oxidized ICWs and mock-treated ICWs were hydrolyzed with 0.1 M glycine-HCl, pH 2.5, 100°C for 30 min after *N*-acetylation, and each resulting supernatant was subjected to chromatography on a DEAE-cellulose column (described in Figure 3). Hexose in each fraction was determined. **(C)** ICWs were treated with a combination of *N*-acetylation, mild acid and mild base according to the flowchart in **(A)**. The supernatant obtained by each mild acid treatment (Sup 1 to 5 in **A**) was subjected to chromatography on a DEAE-cellulose column. Hexose in each fraction was determined. **(D)** Depictions of chemically modified ICWs prepared as shown in **(A)**. Acid-treated ICWs contain only WTA, base-treated ICWs contain only WPS, and acid-treated *N*Ac-ICWs and HF-treated ICW lack both WTA and WPS.

### Analysis of cell wall glycopolymers

Supernatants obtained in each step of mild acid treatment of ICWs were applied to a DEAE-cellulose column (5 ml bed volume) equilibrated with 5 mM Tris–HCl (pH 7.4), and the column was eluted in a stepwise manner with the same buffer containing 50, 150, 250, 350, and 500 mM NaCl, respectively. Each fraction (5 ml) was collected, and hexose and phosphorus in each fraction were determined by the phenol/H_2_SO_4_ method and the method of Lowry et al. (1954), respectively. Fractions eluted at 250 mM NaCl were dialyzed and applied to a DEAE-Sepharose^®^ column (2 × 10 cm) equilibrated with 5 mM Tris–HCl (pH 7.4), and the column was eluted with a linear gradient of NaCl (0–350 mM) in the same buffer. Fractions containing hexose and phosphorus that eluted at about 200–250 mM NaCl were pooled, dialyzed, and subjected to chromatography on a HiPrep™ Sephacryl^®^ S-300 HR column (1.6 × 60 cm, Sigma-Aldrich) in 50 mM (NH_4_)_2_CO_3_. The resultant fractions containing hexose and phosphorus were pooled, dialyzed and used as purified soluble WTAs. To obtain the repeating unit of WTAs, the purified soluble WTA preparation was treated with 47% HF 4°C for 24 h, dried and then subjected to chromatography on a Sephadex G-25 column (1.0 × 80 cm) equilibrated in 50 mM (NH_4_)_2_CO_3_. Fractions containing hexose were pooled and used as the repeating unit of WTAs. For structural analysis of WTAs, ^1^H- and ^13^C-NMR spectra and correlation spectroscopy (COSY), ^1^H-^13^C hetero-nuclear single quantum coherence (HSQC), and ^1^H-^31^P heteronuclear multiple bond connectivity (HMBC) NMR spectra of each purified soluble WTA preparation and its repeating unit were recorded on a Bruker Avance 500 spectrometer in deuterium oxide at room temperature. Chemical shifts were referenced to solvent values (δ 4.70 ppm for HOD). Spectral analyses were performed using TopSpin software ver. 3.6.1. (Bruker BioSpin). Representative ^13^C-NMR spectra of WTAs purified from ICWs were shown in Supplementary Figure 1.

### Smith degradation of intact cell walls

Smith degradation of ICWs was carried out according to the protocol described in the previous report (Misaki and Kanamaru, 1968). Briefly, ICWs (100 mg) were oxidized with 0.1 M NaIO_4_ in 0.1 M sodium acetate buffer (pH 4.5) in the dark at 4°C for 48 h. For mock treatment, ICWs were treated in the same buffer without NaIO_4_. After centrifugation, the respective precipitates were reduced with 0.1 M NaBH_4_ at pH 9.6 for 24 h at 4°C, washed with water, and treated with 0.1 M glycine-HCl buffer (pH 2.5) at room temperature for 1 h. After centrifugation, the precipitates were used as periodate-oxidized ICWs and mock-treated ICWs, respectively. During this process, most hexose and phosphorus in the periodate-oxidized ICWs was eliminated.

### Disruption of intact cell walls

The ICWs (50 mg) were placed in a 1.5-ml tube and disrupted using zirconia beads (0.1 mm Φ, YTZ-0.1, Toso) by intense vortex for 10 min. After centrifugation at 3,000 × *g*, supernatant was collected. The precipitates were further disrupted repeatedly in the same way. The supernatant in each disruption cycle was collected, pooled, and then centrifuged at 12,000 × *g* for 30 min. The resulting precipitates were lyophilized and used as DCWs. During this process, most of the hexose and phosphorus in DCWs was retained.

### Assessment of internalization of intact cell walls by macrophages

The ICWs were labeled with fluorescein using NHS-fluorescein in 50 mM sodium carbonate buffer (pH 9.6) for 30 min at room temperature. Mild acid-, mild base-, and HF-treated fluorescein-labeled ICWs and fluorescein-labeled DCWs were prepared from fluorescein-labeled ICWs, as described above. J774.1 cells (5 × 10^5^/ml) were added in a 1.5-ml high hydrophilic polymer surface treated tube (Proteosave, Sumitomo Bakelite) and incubated with 10 μg/ml of fluorescein-labeled ICWs for 30 min or 24 h at 37°C with gentle rotation. After incubation, fluorescent signals in the cells were analyzed using FACSVerse (BD) after staining with a PerCP-Cy5.5-labeled anti-CD11b monoclonal antibody (BD Pharmingen).

### Statistical analysis

Data are expressed as mean ± SE. Unless otherwise indicated, significance among groups was evaluated by one-way analysis of variance (ANOVA). Values were considered to differ significantly at *P* < 0.05.

## Results

### Effects of intact cell walls prepared from *Lactiplantibacillus plantarum* on cytokine production from macrophages

Intact cell walls were prepared from eight strains of lactobacilli: *L. gasseri* JCM 1131*^T^* and JCM 5814; *L. fermentum* ATCC 9338; *L. helveticus* YIT 0049; *L. plantarum* TUA 5099L, NRIC 1067*^T^*, and YIT 0139; and *L. rhamnosus* ATCC 7469*^T^*. Representative scanning electron micrographs of ICWs isolated from heat-killed whole bacterial cells are shown in Figure 1. The shapes of ICWs of *L. plantarum* TUA 5099L (Figure 1B) are the same as those of the heat-killed cells (Figure 1A). The immunomodulatory properties of ICWs of these strains were assessed using murine macrophage-like J774.1 cells. Stimulation with ICWs prepared from *L. plantarum* TUA 5099L, NRIC 1067*^T^*, and YIT 0139 and *L. helveticus* YIT 0049 elicited secretion of significant levels of IL12 (Figure 2), whereas secretion of the cytokine from cells stimulated with ICWs from strains of *L. rhamnosus, L. fermentum*, and *L. gasseri* was almost negligible. These results were reproducible in stimulation of murine BMMs with the respective ICWs (Figure 2).

### Analyses of cell wall polysaccharide components in intact cell walls

To investigate involvement of cell wall glycopolymers in the immunomodulatory properties of ICWs, we next analyzed the glycopolymers on the ICWs. Cell wall glycopolymers of Gram-positive bacteria are attached to the 6-position of muramic acid residues in peptidoglycans through an acid labile sugar-1-phosphate linkage (Weidenmaier and Peschel, 2007), and thus, can be released from peptidoglycans by mild acid treatment. Therefore, ICWs were first *N*-acetylated and then the resulting *N-*acetylated ICWs (*N*Ac-ICWs) were treated with 0.1 M glycine-HCl buffer (pH 2.5) at 100°C for 30 min, and the resulting supernatants were separated in a stepwise manner using a DEAE-cellulose column.

Mild acid hydrolysis of *N*Ac-ICWs of *L. plantarum* TUA 5099L gave two hexose and phosphorus-containing polymers; one was eluted from the column with 150 mM NaCl and the other with 250 mM NaCl (Figure 3A). Since *L. plantarum* TUA 5099L have been shown to express WTAs (Tomita et al., 2012) and the typical WTAs of various Gram-positive bacteria [i.e., poly(glycerol phosphate), poly(ribitol phosphate), and poly(glycosylglycerol phosphate)] are eluted from a DEAE-cellulose column at 200–300 mM NaCl (Kaya et al., 1984; Kojima et al., 1985a), the hexose and phosphate-containing polymers eluted with 250 mM NaCl were predicted to be WTAs. Based on NMR data of the purified WTA (Supplementary Table 1) and its repeating unit, this glycopolymer was determined to be poly(6-kojibiosyl-α1-glycerol 3-phosphate), as reported previously (Tomita et al., 2012). The detailed structure of the glycopolymer eluted at 150 mM NaCl was not determined, but this glycopolymer was a kind of acidic cell wall polysaccharide (WPS).

Mild acid hydrolysis of *N*Ac-ICWs of *L. plantarum* NRIC 1067*^T^* and YIT 0139 and those of *L. helveticus* YIT 0049 also gave phosphate-containing glycopolymers eluted with 250 mM NaCl (Figure 3B), which were determined to be poly(1-(2- and 4-α-diglucosyl)ribitol 5-phosphate), poly(1-(2- or 4-α-monoglucosyl)ribitol 5-phosphate), and poly(1-glycerol 3-phosphate), respectively, from NMR data of the respective purified WTAs (Supplementary Table 1) and their repeating units. Therefore, the ICWs of *L. plantarum* TUA 5099L, NRIC 1067*^T^* and YIT 0139, and those of *L. helveticus* YIT 0049 displayed typical WTAs on their surfaces. These WTAs did not contain ester-linked D-alanine residues because a signal for the methyl group of D-alanine (around 17 ppm) was not detected in ^13^C-NMR spectra. In contrast, ICWs of *L. rhamnosus, L. fermentum*, and *L. gasseri*, which did not elicit IL-12 production, did not contain the glycopolymers eluted at 250 mM NaCl (Figure 3B), indicating that typical WTAs are not present on the cell walls of these strains. In fact, expression of WTAs on the *L. gasseri, L. fermentum*, and *L. rhamnosus* strains have not been reported (Kleerebezem et al., 2010). Thus, these results suggest that the presence of typical WTAs on ICWs is required for elicitation of IL-12 production from macrophages by ICWs of lactobacilli.

### Wall teichoic acids displayed on intact cell walls of *Lactiplantibacillus plantarum* are required for IL-12 production from macrophages

**FIGURE 5:**
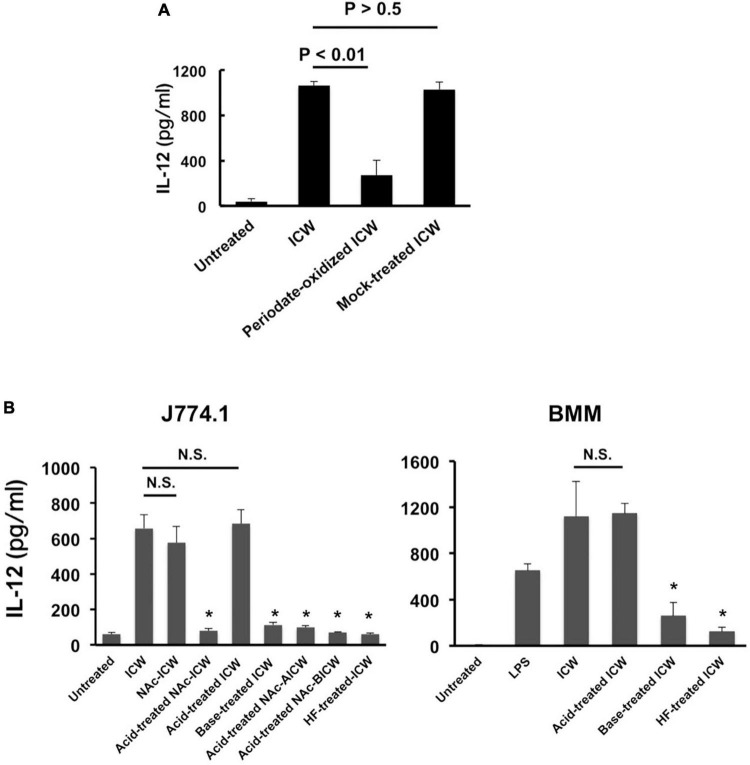
IL-12 secretion from macrophages stimulated with chemically modified ICWs of *L. plantarum* TUA 5099L. **(A)** J774.1 cells were cultured with unmodified, periodate-oxidized or mock-treated ICWs (each 10 μg/ml) for 24 h and IL-12 secreted in each supernatant was determined. Each bar is the mean ± SE for three independent experiments. **(B)** J774.1 cells or BMMs were cultured with chemically modified or unmodified ICWs (10 μg/ml) for 24 h and IL-12 secreted in the culture supernatant was determined. Each bar is the mean ± SE for 4 (for J774.1 cells) and 3 (for BMMs) experiments. Significance was evaluated by one-way ANOVA. N.S., not significant vs. ICW treated cells. *P < 0.01 vs. cells treated with unmodified ICWs.

To evaluate roles of WTAs in IL-12 secretion from macrophages upon stimulation with ICWs of *L. plantarum* TUA 5099L, various types of chemically modified ICWs were prepared. The procedures for sequential chemical modifications of ICWs are summarized in Figure 4A. Vicinal diols-containing carbohydrates and alditols can be oxidatively cleaved by sodium periodate (NaIO_4_). Smith degradation (periodate oxidation, NaBH_4_ reduction followed by mild acid hydrolysis) (Abdel-Akher et al., 1952) is an approach to selective and specific elimination of cell wall glycopolymers, including WTAs. As shown in Figure 4B, periodate-oxidized ICWs of *L. plantarum* TUA 5099L did not give WTAs or WPSs after ICWs were *N*-acetylated and then treated with mild acid, indicating that NaIO_4_ oxidation leads to degradation of both WTAs and WPSs on ICWs. Furthermore, IL-12 induction by the periodate-oxidized ICWs was reduced significantly compared to that for unmodified ICWs (Figure 5A). In contrast, mock treatment without NaIO_4_ oxidation did not affect both glycopolymers and IL-12 induction by ICWs. These results indicate that the glycopolymers on ICWs are essential for IL-12 induction by ICWs of *L. plantarum* TUA 5099L.

Wall teichoic acids (WTAs) (but not WPSs) of some strains of *L. plantarum* are not easily released from cell wall peptidoglycans by mild acid treatment (0.1 M glycine-HCl buffer, pH 2.5, 100°C for 30 min), but are released by mild base (0.5 M NaOH at 37°C for 30 min) (Kojima et al., 1985b). Therefore, WTAs and other glycopolymers in ICWs of *L. plantarum* can be selectively removed from ICWs by a combination of mild acid and mild base treatments. Mild acid treatment of the *N*-acetylated ICWs (*N*Ac-ICWs) gave both WTAs (250 mM NaCl eluates) and WPSs (150 mM NaCl eluates) in supernatants (Sup 1 in Figure 4C). Treatment of the resulting acid-treated *N*Ac-ICWs with further mild acid released neither WTAs nor WPSs (Sup 2 in Figure 4C), indicating that acid-treated *N*Ac-ICWs no longer display both glycopolymers. The same mild acid treatment of unmodified ICWs gave only WPSs that eluted at 150 mM NaCl (Sup 3 in Figure 4C). Treatment of the AICWs again with mild acid after *N*-acetylation resulted in release of WTAs that eluted at 250 mM NaCl (Sup 4 in F Figure 4C). On the other hand, initial treatment of ICWs with mild base followed by treatment of the resulting BICWs with mild acid after *N*-acetylation gave only WPSs that eluted at 150 mM NaCl (Sup 5 in Figure 4C). These results indicated that only WTAs of two types of glycopolymers were retained in AICWs, while only WPSs were held in BICWs (see schematic illustration in Figure 4D). Since aqueous HF was used to gently cleave phosphodiester bonds, HF-treated ICWs did not contain both WTAs and WPSs. The acid- and BICWs both retained their particulate shapes (Figures 1C,D).

The immunomodulatory properties of these chemically modified ICWs were assessed based on IL-12 secretion from J774.1 cells. Stimulation of the cells with acid- treated *N*Ac-ICWs and HF-treated-ICWs did not induce IL-12 secretion (Figure 5B, left panel). Purified soluble WTA (500 μg/ml) and purified soluble WPS (500 μg/ml) also failed to induce IL-12 secretion (Supplementary Figure 2). However, AICWs did induce IL-12, similarly to unmodified ICWs (Figure 5B, left panel). In contrast, BICWs failed to elicit IL-12 secretion, similarly to acid-treated *N*Ac-ICWs and HF-treated ICWs. AICWs also induced significant levels of IL-12 from BMMs, while induction by base-treated and HF-treated ICWs was markedly diminished compared to that of unmodified ICWs (Figure 5B, right panel). Similar results were obtained with treatment of ICWs of *L. plantarum* NRIC 1067*^T^* and YIT 0139 with mild acid and base (Supplementary Figure 3). As the unmodified, acid-treated, and mock-treated ICWs display WTAs, while the base-treated, HF-treated and periodate-oxidized ICWs lack surface WTAs, our results strongly indicate that WTAs displayed on ICWs play important roles in the immunomodulatory properties of ICWs.

### Intact cell wall-mediated IL-12 secretion is elicited through a TLR2 signaling independent pathway

**FIGURE 6:**
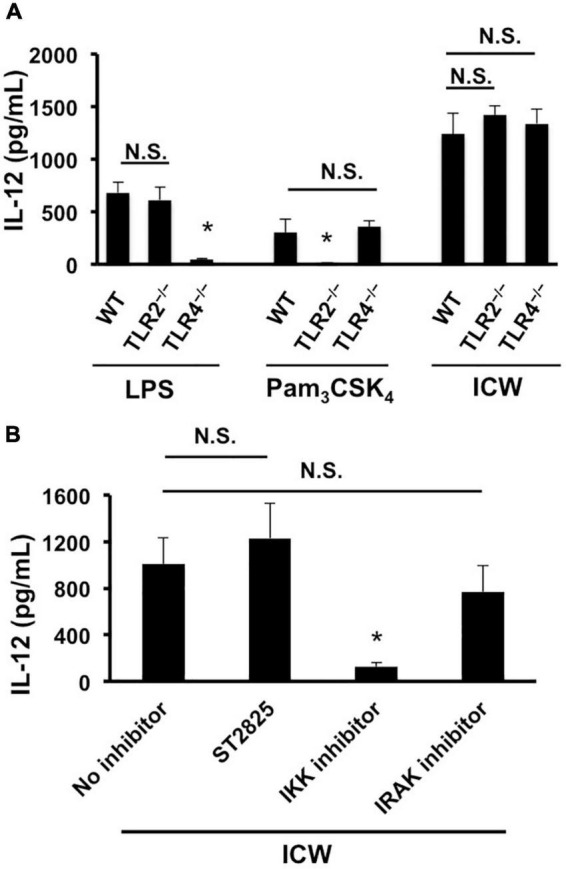
Involvement of TLRs in IL-12 secretion from macrophages stimulated with ICWs. **(A)** BMMs were prepared from wild-type (WT), TLR2-deficient (TLR2^–/–^), and TLR4-deficient (TLR4^–/–^) mice, respectively. BMMs (5 × 10^4^/ml) were cultured with 1 μg/ml of Pam_3_CSK_4_, 500 ng/ml of LPS, or 10 μg/ml of ICWs for 24 h and IL-12 secreted in the culture supernatant was determined. Each bar is the mean ± SE for three independent experiments. N.S., not significant vs. BMMs from wild-type mice. *P < 0.01 vs. cells from wild-type mice. **(B)** BMMs prepared from wild-type mice were pretreated with 10 μM of IKK inhibitor II, 10 μM of ST2825, a MyD88 dimerization inhibitor and 0.5 μM of IRAK1/4 inhibitor I, respectively, for 30 min, and then cells were stimulated with 10 μg/ml of ICWs. After 24 h stimulation, IL-12 secreted in the culture supernatant was determined. Each bar is the mean ± SE for three independent experiments. N.S., not significant vs. cells treated without inhibitors (no inhibitor).

It has been demonstrated that the immunomodulatory properties of *L. plantarum* are triggered by recognition of cell wall components such as LTAs with TLR2 (Matsuguchi et al., 2003; Hirose et al., 2010). We therefore examined the involvement of TLR2 signaling in IL-12 induction from macrophages stimulated with ICWs of *L. plantarum* TUA 5099L. BMMs prepared from a TLR2-deficient mouse secreted IL-12 upon ICW stimulation similar to those from a wild-type mouse, although they failed to induce IL-12 in response to stimulation with Pam_3_CSK_4_, a TLR2 agonist (Figure 6A). BMMs deficient in TLR4 also induce IL-12 in response to ICW stimulation. ICW-mediated IL-12 secretion from BMMs was clearly inhibited by 10 μM IKK inhibitor II that inhibit IKKs α and β, but was not affected by 10 μM ST2825, which is a specific MyD88 dimerization inhibitor and interferes with recruitment of IRAK1/4 by MyD88, (Loiarro et al., 2007), and 0.5 μM IRAK 1/4 inhibitor I (Figure 6B). These results indicate that ICW-mediated IL-12 secretion from macrophages is elicited through TLR2-MyD88-IRAK1/4 signaling axis independent pathway.

### Wall teichoic acids displayed on intact cell walls are essential for rapid uptake of intact cell walls by macrophages

**FIGURE 7:**
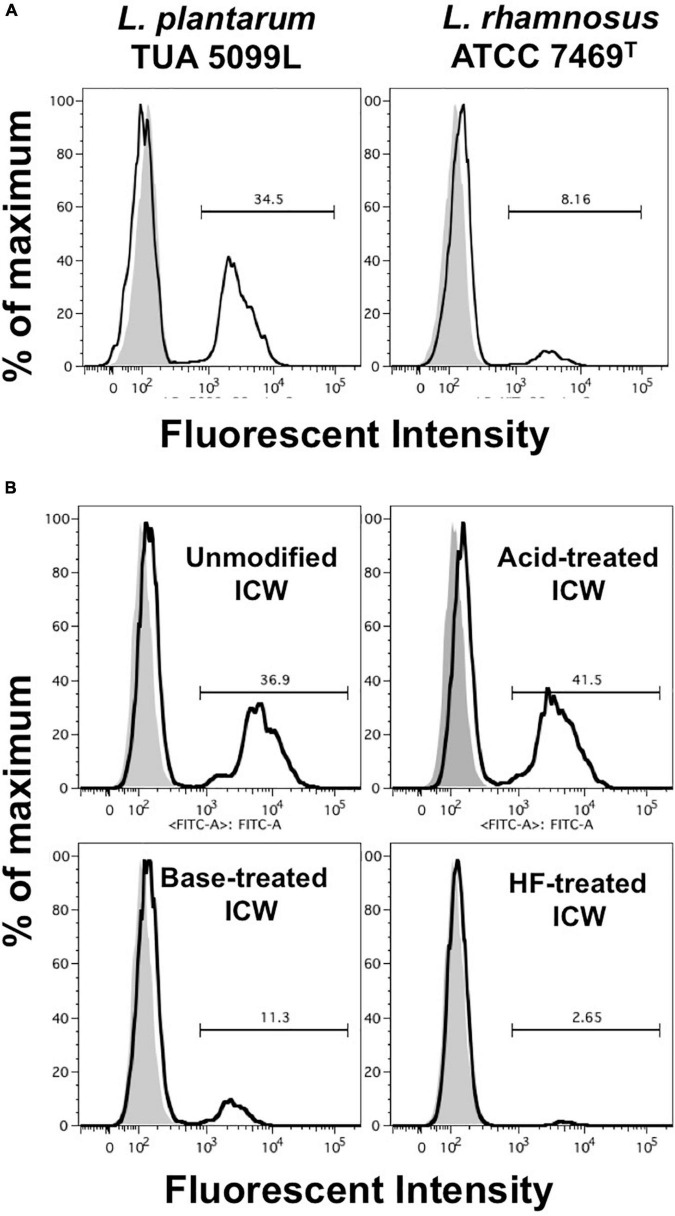
Uptake of ICWs into macrophages. **(A)** J774.1 cells were incubated with fluorescein-labeled WTA-displaying ICWs of *L. plantarum* TUA 5099L and WTA-lacking ICWs of *L. rhamnosus* ATCC 7496*^T^* for 30 min at 37°C with gentle rotation. Cells were washed with PBS and fluorescent signals in the cells were then analyzed by flow cytometry. Representative results of two independent experiments are shown. Gray and open histograms indicate untreated and fluorescein-labeled ICWs treated cells, respectively. **(B)** J774.1 cells were incubated with chemically modified ICWs prepared from fluorescein-labeled ICWs of *L. plantarum* TUA 5099 for 30 min and fluorescent signals in the cells were analyzed. Representative results of two independent experiments are shown. Gray and open histograms indicate untreated and fluorescein-labeled ICWs treated cells, respectively.

As uptake of bacteria and recognition of bacterial components are important in induction of proinflammatory cytokines, we assessed uptake of fluorescein-labeled ICWs into J774.1 cells by means of a flow cytometer. After incubation for 30 min with fluorescein-labeled WTA-displaying ICWs of *L. plantarum* TUA 5099L, about 40% of the cells had a strong fluorescent signal (Figure 7A). In contrast, <10% of the cells showed fluorescent signals after incubation with fluorescein-labeled WTA-lacking ICWs of *L. rhamnosus*, suggesting that WTAs displayed on ICWs are needed for cellular recognition and uptake of ICWs. To confirm this postulate, uptake of fluorescein-labeled acid- or BICWs of *L. plantarum* TUA 5099L was investigated. WTA-displaying AICWs were ingested into cells within 30 min to almost the same extent as unmodified ICWs (Figure 7B), whereas WTA-lacking base-treated and HF-treated ICWs were barely ingested over 30 min. Therefore, WTAs displayed on ICWs are required for rapid recognition and uptake of ICWs and subsequent IL-12 production by J774.1 cells.

### Loss of IL-12 induction by intact cell walls with inhibition of actin remodeling

**FIGURE 8:**
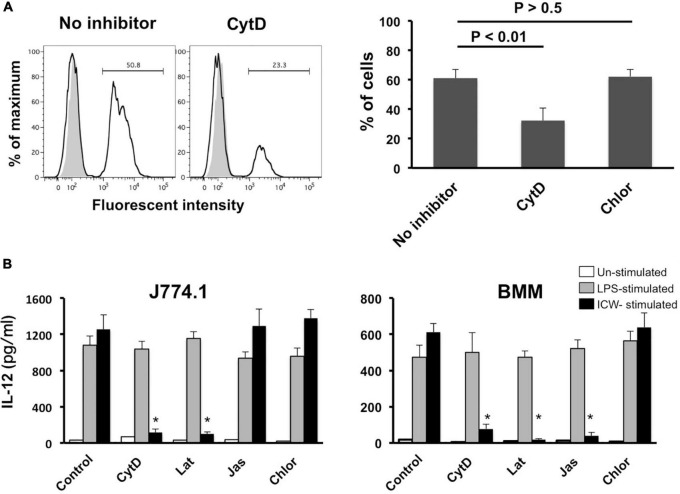
Effect of actin polymerization inhibitors on uptake of ICWs and IL-12 secretion. **(A)** J774.1 cells were treated with cytochalasin D (CytD, 1 μM) or chlorpromazine (Chlor, 1 μg/ml) in complete RPMI1640 containing 0.1% DMSO for 30 min at 37°C. Fluorescein-labeled ICWs of *L. plantarum* TUA 5099L were added and the mixture was incubated for 30 min. Fluorescent signals in the cells were analyzed. Representative histograms (left) and percentages of positive cells among the treated cells (right) are shown. Gray and open histograms indicate untreated and fluorescein-labeled ICWs treated cells, respectively. **(B)** J774.1 cells and BMMs were treated with CytD (1 μM), latrunculin B (Lat, 1 μM), jasplakinolide (Jas, 0.1 μM), or Chlor (1 μg/ml) in complete RPMI1640 containing 0.1% DMSO for 30 min. Then the cells were stimulated with ICWs of *L. plantarum* TUA 5099L (10 μg/ml) or LPS (100 ng/ml). Culture supernatants were collected after 24 h and IL-12 in supernatants was determined. Open, gray and black columns indicate unstimulated, LPS-stimulated, and ICW-stimulated cells, respectively. Each bar is the mean ± SE for four independent experiments. *P < 0.01 vs. inhibitor-untreated cells.

It is well accepted that particles of diameter >500 nm are ingested into mononuclear phagocytes by phagocytosis (Aderem and Underhill, 1999; Uribe-Querol and Rosales, 2020), while those of diameter <500 nm are taken up by clathrin-based receptor-mediated endocytosis. As phagocytosis is actin-dependent, we examined the role of phagocytosis of ICWs in induction of IL-12 secretion using inhibitors of actin polymerization. Treatment with cytochalasin D, an actin polymerization inhibitor, markedly reduced rapid uptake of ICWs into J774.1 cells (Figure 8A). In contrast, treatment with chlorpromazine, a clathrin-mediated endocytic inhibitor, did not affect uptake of the ICWs. Treatment of J774.1 cells and BMMs with cytochalasin D led to a marked reduction of IL-12 production from these cells after ICW stimulation, whereas IL-12 elicited by LPS was not affected by the same treatment (Figure 8B). Latrunculin B, another actin polymerization inhibitor, also inhibited IL-12 production from both cell types, whereas chlorpromazine did not affect cytokine production. It should be noted that IL-12 production from BMMs was also abolished by treatment with jasplakinolide, an inhibitor of actin depolymerization (Figure 8B), whereas this treatment did not affect IL-12 production in J774.1 cells. As a higher concentration of jasplakinolide showed strong cell toxicity, we did not test the effect of this higher concentration on cytokine secretion.

### Loss of IL-12 induction by intact cell walls with disrupted shapes

**FIGURE 9:**
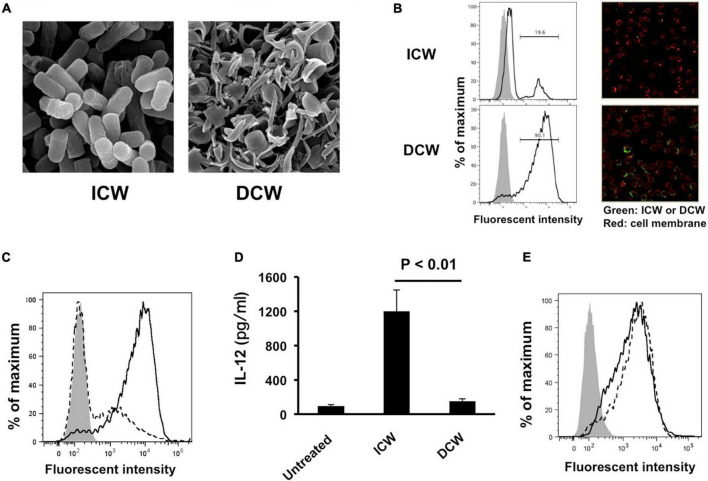
Effects of disruption of overall shapes of ICWs on immunomodulatory abilities of ICWs. **(A)** Scanning electron microscopy of ICWs and DCWs of *L. plantarum* TUA 5099L. **(B)** Uptake of ICWs or DCWs. J774.1 cells were incubated with fluorescein-labeled ICWs or disrupted ICWs (DCWs) of *L. plantarum* TUA 5099 for 30 min and fluorescent signals in the cells were analyzed. Gray and open histograms indicate untreated and fluorescein-labeled cell walls treated cells, respectively. Representative images under florescent microscopy were also shown. **(C)** Reduction of uptake of DCWs by HF treatment. J774.1 cells were incubated with fluorescein-labeled DCWs (solid line) or HF-treated DCWs (dashed line) for 30 min and fluorescent signals in the cells were analyzed. Gray histograms indicate cells without DCW treatment. **(D)** Elicitation of IL-12 secretion from macrophages. J774.1 cells were cultured with ICWs (10 μg/ml) or DCWs (100 μg/ml) for 24 h and IL-12 secreted in culture supernatants was determined. Each bar is the mean ± SE for three independent experiments. **(E)** Effect of cytochalasin D on uptake of DCWs. J774.1 cells were treated with cytochalasin D in complete RPMI1640 containing 0.1% DMSO for 30 min at 37°C. Fluorescein-labeled DCWs were added and the mixture was incubated for 30 min. Fluorescent signals in the cells were analyzed. Solid and dashed lines indicate untreated and cytochalasin D-treated cells, respectively. Gray histograms indicate cells without DCW treatment.

**FIGURE 10:**
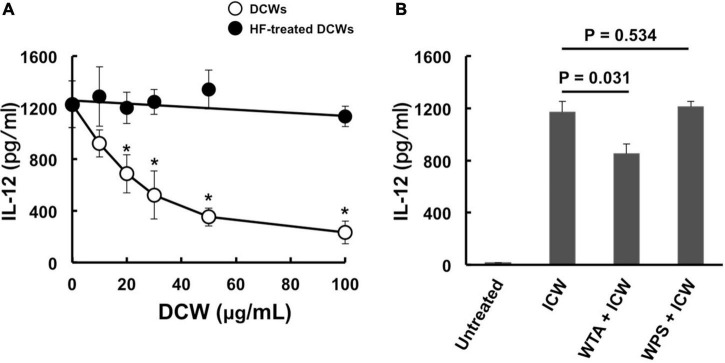
Inhibition of immunomodulation by ICWs by pretreatment of cells with DCWs and soluble WTAs. **(A)** J774.1 cells were treated with various concentrations of DCWs of *L. plantarum* TUA 5099L (open circles) or HF-treated DCWs (closed circles) for 30 min at 37°C with gentle rotation. Then the cells were cultured with 10 μg/ml of ICWs of *L. plantarum* TUA 5099L for 24 h, and IL-12 secreted in culture supernatants was determined. Each bar is the mean ± SE for three independent experiments. *P < 0.01 vs. DCW-untreated cells. **(B)** Cells were treated with 500 μg/ml of purified soluble WTAs or WPS of *L. plantarum* TUA 5099L for 30 min at 37°C. Then the cells were stimulated with 10 μg/ml of ICWs of *L. plantarum* TUA 5099L for 24 h, and IL-12 secreted in culture supernatants was determined. Each bar is the mean ± SE for three independent experiments.

Physical disruption of ICWs using zirconia beads caused the ICWs to become fragmented, and the overall shapes of the disrupted ICWs (DCWs) became fibrous (Figure 9A). DCWs were incorporated into J774.1 cells more effectively than ICWs within 30 min, and HF treatment of DCWs caused marked reduction of uptake (Figures 9B,C), but DCWs (100 μg/ml) failed to induce IL-12 secretion from J774.1 cells and BMMs (Figure 9D). Uptake of DCWs was not affected by cytochalasin D (Figure 9E), suggesting that DCWs are taken up in an actin-independent manner. However, DCWs, but not HF-treated DCWs, reduced IL-12 secretion from ICW-stimulated J774.1 cells in a DCW concentration-dependent manner (Figure 10A). In addition, pretreatment of cells with purified soluble WTA (500 μg/ml) led to a significant reduction of IL-12 secretion from ICW-stimulated cells, whereas pretreatment with WPS (500 μg/ml) did not affect IL-12 secretion (Figure 10B).

## Discussion

The effects of WTAs of lactic acid bacteria on immunomodulation remain unclear, although LTAs of lactic acid bacteria are recognized as major cell wall components to elicit proinflammatory cytokine production. One of the aims of this study is to clarify immunomodulatory activities of WTAs attached to peptidoglycan. Here, we showed the importance of WTAs in eliciting IL-12 production from murine macrophages stimulated with ICWs of *L. plantarum*. A requirement for WTAs for IL-12 induction by ICWs was confirmed by the following evidences: (i) WTA-displaying ICWs of *L. plantarum* and *L. helveticus* induced IL-12 production from J774.1 murine macrophage-like cells and BMMs, whereas WTA-lacking ICWs of *L. gasseri*, *L. fermentum*, and *L. rhamnosus* did not do so; (ii) selective elimination of WTAs from ICWs of *L. plantarum* TUA 5099L, but not of WPSs other than WTAs, abolished IL-12 induction; and (iii) soluble WTAs, but not WPSs, purified from ICWs of *L. plantarum* TUA 5099L inhibited IL-12 secretion by ICW stimulation. ICW-mediated IL-12 secretion is closely related to WTA-mediated rapid internalization of ICWs into the cells, since WTA-displaying ICWs, but not WTA-lacking ICWs, were rapidly recognized and engulfed by macrophages.

Most WTAs of Gram-positive bacteria are attached to muramic acid residues in peptidoglycans through a common linkage unit, glycerophosphoryl-*N*-acetylmannosaminyl(β1-4) *N*-acetylglucosamine (Gro-P-ManNAcβ1-4GlcNAc) *via* an acid labile GlcNAc 1-phosphate linkage (Araki and Ito, 1989), and thus, mild acid hydrolysis (pH 2.5, 100°C, 30 min) releases WTAs from cell walls (Kaya et al., 1984; Kojima et al., 1985a). However, WTAs of *L. plantarum* are attached through a unique unit, glycerophosphoryl-*N*-acetylmannosaminyl (β1-4)glucosamine (Gro-P-ManNAcβ1-4GlcN), *via* a GlcN 1-phosphate linkage that is barely cleaved by mild acid hydrolysis due to the effect of the free amino group limiting acid hydrolysis of a hexaminide, and thus WTAs in *L. plantarum* exhibit anomalous acid stability, and only WPSs are released from peptidoglycans by mild acid hydrolysis (Kojima et al., 1985b). After the GlcN in the linkage is converted to GlcNAc by *N*-acetylation, both WTAs and WPSs are released from the cell walls by mild acid treatment. In contrast, WTAs, but not WPSs, in *L. plantarum* strains are easily eliminated from cell walls by mild base treatment (0.5 M NaOH, 37°C, 30 min) because phosphodiester bonds of glycerol phosphate in the linkage unit of WTAs are labile with mild base (Kojima et al., 1985a,b). These findings led us to the idea that a combination of mild acid treatment, mild base treatment, and *N*-acetylation of ICWs of *L. plantarum* could be used for selective removal of WTAs and/or WPSs from ICWs. Using this approach, we were able to prepare ICWs displaying only WTAs, only WPSs, or lacking both glycopolymers from unmodified ICWs of *L. plantarum* TUA 5099L (Figure 4D). We then showed that WTA-lacking ICWs could not induce IL-12 production in murine macrophages, while those displaying WTAs retained this ability.

Mild base treatment and HF treatment also affects nucleic acids and proteins, which makes it possible that IL-12 secretion stimulated by ICWs is caused by contamination of these molecules in the ICW preparations. However, this possibility was ruled out by Smith degradation of ICWs. Smith degradation leads to oxidative cleavage of vicinal diols in carbohydrates and alditols in glycopolymers, but does not affect peptidoglycans, proteins, and nucleic acids because they do not contain vicinal diols in their backbone structures. As expected, Smith degradation of ICWs of *L. plantarum* TUA 5099L led to degradation of both WTAs and WPSs on ICWs and marked reduction of IL-12 production. Involvement of LTAs, a TLR2 agonist, in IL-12 production by ICWs is also excluded if LTAs are contaminants in ICW preparations from following reasons: (i) LTAs are expressed in most lactobacilli, including *L. gasseri, L. fermentum*, and *L. rhamnosus* (Kleerebezem et al., 2010; Shiraishi et al., 2016); and (ii) BMMs prepared from TLR2-deficient mice could secret almost the same levels of IL-12 as those from wild-type mice upon stimulation with ICWs of *L. plantarum*. In addition, a MyD88 dimerization inhibitor, ST2825, and an IRAK inhibitor did not affect the ICW-mediated IL-12 secretion. Taken together, these results clearly indicate that cell wall glycopolymers, and particularly WTAs, on ICWs of *L. plantarum* play essential roles in ICW-mediated IL-12 secretion from murine macrophages, which is elicited through a TLR2-MyD88-IRAK axis independent signaling process.

We also showed in this paper that macrophages secreted IL-12 in response to ICW stimulation in an actin remodeling dependent and/or phagocytosis dependent manner. Treatment with cytochalasin D and latrunculin B abolished ICW-mediated IL-12 production, and clearly diminished rapid internalization of ICWs into the cells, indicating that WTA-mediated phagocytosis of ICWs may be required for elicitation of IL-12 production from cells stimulated with ICWs. Treatment of BMMs with jasplakinolide, an actin filament polymerizing and stabilizing agent, also inhibited ICW-mediated IL-12 production. Several lines of evidence indicate that cytokine production from mononuclear phagocytes is regulated by phagocytic processes of microbes and related particles, including ICWs, as cytokine production from these cells is influenced by cytochalasin D (Naruse et al., 2011; Camilli et al., 2018). Phagocytosis of microbes leads to their intracellular digestion, and the resulting digest can elicit cytokine production through recognition of pathogen-associated molecular patterns (PAMPs) in the digests by pattern-recognition receptors in the endosome or cytosol (Naruse et al., 2011; Yoshikawa et al., 2021). However, Shida et al. (2006) found that phagocytosis of ICWs of *Lactobacillus casei* elicited IL-12 production from peritoneal exudative macrophages, and concluded that resistance of the cell wall to intracellular digestion is a critical factor determining the ability of lactobacilli to induce IL-12. In addition, large curdlan particles, which are hardly internalized, can also induce cytokine production from bone marrow-derived dendritic cells (BMDCs) in an actin polymerization-dependent manner (Rosas et al., 2008). We have shown that OMLs with a diameter of 1 μm can elicit IL-12 secretion from mononuclear phagocytes (Takagi et al., 2007), in a process that relies on carbohydrate-dependent engulfment of OMLs accompanied with actin remodeling, although OMLs do not contain any molecules related to typical PAMPs. Indeed, activation of mononuclear phagocytes and IL-12 production induced by OMLs occurs independently of TLR2/4 and MyD88 (Matsuoka et al., 2016, 2019). These observations indicate that a phagocytic process and subsequent intracellular digestion and recognition of PAMPs may not be absolutely necessary to elicit IL-12 production by stimulation with carbohydrate-decorated particles.

Phagocytosis is a cellular process to ingest and eliminate particles over 500 nm in diameter (Aderem and Underhill, 1999; Uribe-Querol and Rosales, 2020). We have shown recently that mononuclear phagocytes treated with OMLs of diameter ≥600 nm can secrete IL-12, while those treated with OMLs of ≤400 nm fail to produce this cytokine, although the cells are activated by both sizes of OMLs (Matsuoka et al., 2021). In the current study, we showed that DCWs of *L. plantarum* failed to elicit IL-12 production. This result is consistent with the previous observation that disruption of ICWs of *Bifidobacterium infantis* reduced the anti-tumor activity of ICWs (Sekine et al., 1985). DCWs of *L. plantarum* had fibrous shapes with a 450-nm mean diameter, whereas ICWs had particulate shapes with a 870-nm mean diameter. DCWs were recognized and engulfed by cells more efficiently than ICWs, and recognition and internalization of DCWs clearly depended on WTAs, but the DCWs no longer induced actin remodeling and IL-12 production, since cytochalasin D did not inhibit internalization of DCWs. In addition, internalization of DCWs was not inhibited by treatment with chlorpromazine, a clathrin-mediated endocytic inhibitor. In addition to the classical clathrin-dependent mechanism of endocytosis, there are several clathrin-independent endocytic pathways, i.e., caveolin/dynamin-dependent and caveolin/dynamin-independent pathways (Mayor and Pagano, 2007). Although these clathrin-independent pathways are sometimes used by bacteria and viruses to gain access to the host cell, we could not identify the pathways for internalization of DCWs. However, DCWs markedly inhibited IL-12 secretion from ICW-stimulated cells, and soluble WTAs partially inhibited ICW-mediate IL-12 secretion. Therefore, ICWs and DCWs share the same receptors or binding sites on cells that recognize WTAs, although DCWs and soluble WTAs could not elicit actin remodeling and IL-12 secretion. These findings suggest that the sizes and shapes of ICWs have an impact on actin remodeling and subsequent IL-12 production in mononuclear phagocytes. Recognition of a certain density and orientation of WTAs displayed on ICWs by macrophages may be needed for formation of molecular assemblies on the cells so-called “phagocytic synapse” (Niedergang et al., 2016) like immunological synapse (Bromley et al., 2001) or glycosynapse (Hakomori, 2002) that perform actin remodeling and induction of IL-12. Fragmentation of cell walls may reduce both the integration and orientation of WTAs on cell walls, which may cause failure to form the assemblies. In fact, Li et al. (2020) have demonstrated that the spatial organization of ligand clusters is needed to signal for immune regulation by macrophages. Thus, the sizes and shapes of the ICWs may have an impact on formation of “phagocytic synapse” and subsequent actin remodeling and IL-12 production from macrophages.

## Conclusion

In conclusion, we showed in this study that WTA-displaying ICWs of several strains of *L. plantarum* elicit IL-12 secretion from murine macrophages through an actin remodeling dependent but a TLR2-MyD88-IRAK axis independent process. WTAs displayed on cell walls with preserved bacterial shapes and intact peptidoglycan structures are key molecules in elicitation of actin remodeling and subsequent IL-12 secretion from macrophages upon stimulation with the ICWs. Although limited lines of evidence suggest the importance of cell wall glycopolymers in the immunomodulatory properties of bacteria (Shida et al., 2006; Gorska et al., 2014), this is the first study to show that WTAs of lactobacilli are actually involved in proinflammatory cytokine production from macrophages. However, unlike LTAs, WTA itself has no immunomodulatory activity. The receptors for WTAs displayed on ICWs and the underlying mechanisms of elicitation of actin remodeling and subsequent IL-12 production by ICWs remain unclear. Although TLR signaling is not involved in ICW-mediated IL-12 secretion from macrophages, an alternative upstream signaling cascade that accompanied with actin remodeling is needed for activation of NF-κB, since an IKK inhibitor almost completely inhibits the IL-12 secretion. Structural analyses of WTAs of *L. plantarum* strains suggested that differences in the backbone structures of the WTAs are not involved in this phenomenon. WTAs have many negative charges due to their backbone phosphates, and cells may recognize large clusters of negative charges displayed on ICWs as a molecular pattern. C-type lectin receptors (CLRs) and scavenger receptors expressed on macrophages are the members of pattern recognition receptors, and act as non-opsonic phagocytic receptors for microbes (Uribe-Querol and Rosales, 2020), and mouse SIGNR3, and dectin-2, both of which recognize mannose residues, can bind cell surface components of lactobacilli and mediate phagocytosis of bacteria (Lightfoot et al., 2015; Yoshikawa et al., 2021), and signal through immunoreceptor tyrosine-based activation motifs (Tanne et al., 2009; Brown et al., 2018). Therefore, recognition of WTAs on ICWs by these receptors may trigger the subsequent actin remodeling process. Our preliminary experiments show that ICW-mediated IL-12 secretion from BMMs was inhibited by several monosaccharides (e.g., mannose) in a dose-dependent manner (personal observation), suggesting that some kinds of CLRs are involved in this phenomenon. Identification of the receptors for ICWs of *L. plantarum* may help with understanding of why and how these ICWs exhibit immunomodulatory activities. Further studies aimed at identification of the receptors and signaling pathways are currently in progress.

## Data availability statement

The raw data supporting the conclusions of this article will be made available by the authors, without undue reservation.

## Ethics statement

The animal study was reviewed and approved by the Animal Committee at Tokai University.

## Author contributions

NK, SK, SH, DZ, EO, and YT performed the experiments and purification of WTAs. YO performed the NMR analyses. NK and YK designed the experiments and wrote and edited the manuscript. S-IY and MN provided critical materials and expertise for this research, and reviewed this manuscript. All authors have read and agreed to the submission of the manuscript.
