# Supplementary figures and images for: Wall teichoic acid-dependent phagocytosis of intact cell walls of Lactiplantibacillus plantarum elicits IL-12 secretion from macrophages
Source: Front Microbiol. 2022 Aug 9;13:986396. doi: 10.3389/fmicb.2022.986396 (PMC9396385; doi:10.3389/fmicb.2022.986396)

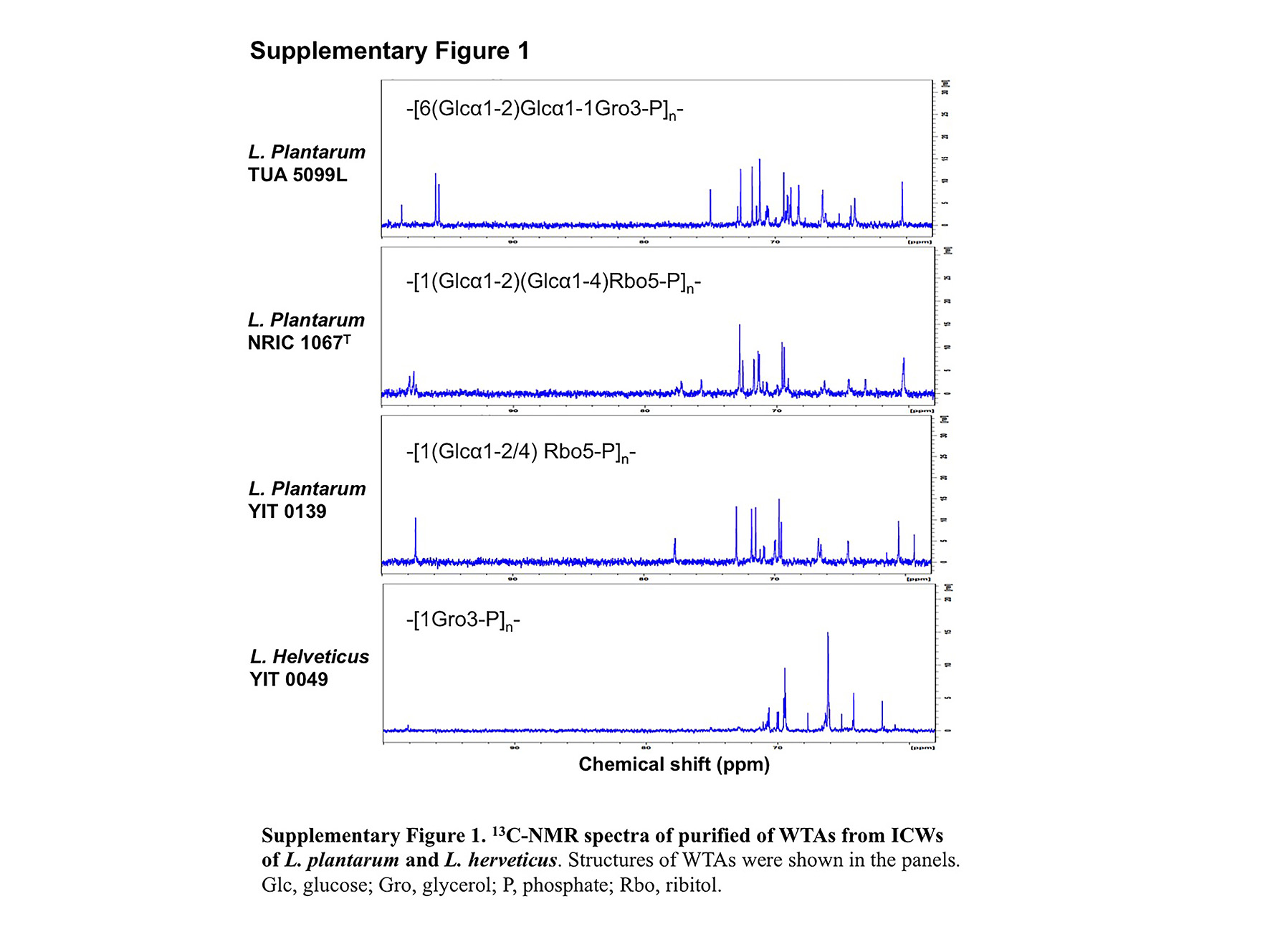

Supplement: Supplementary file 1 [file Image_1.JPEG]

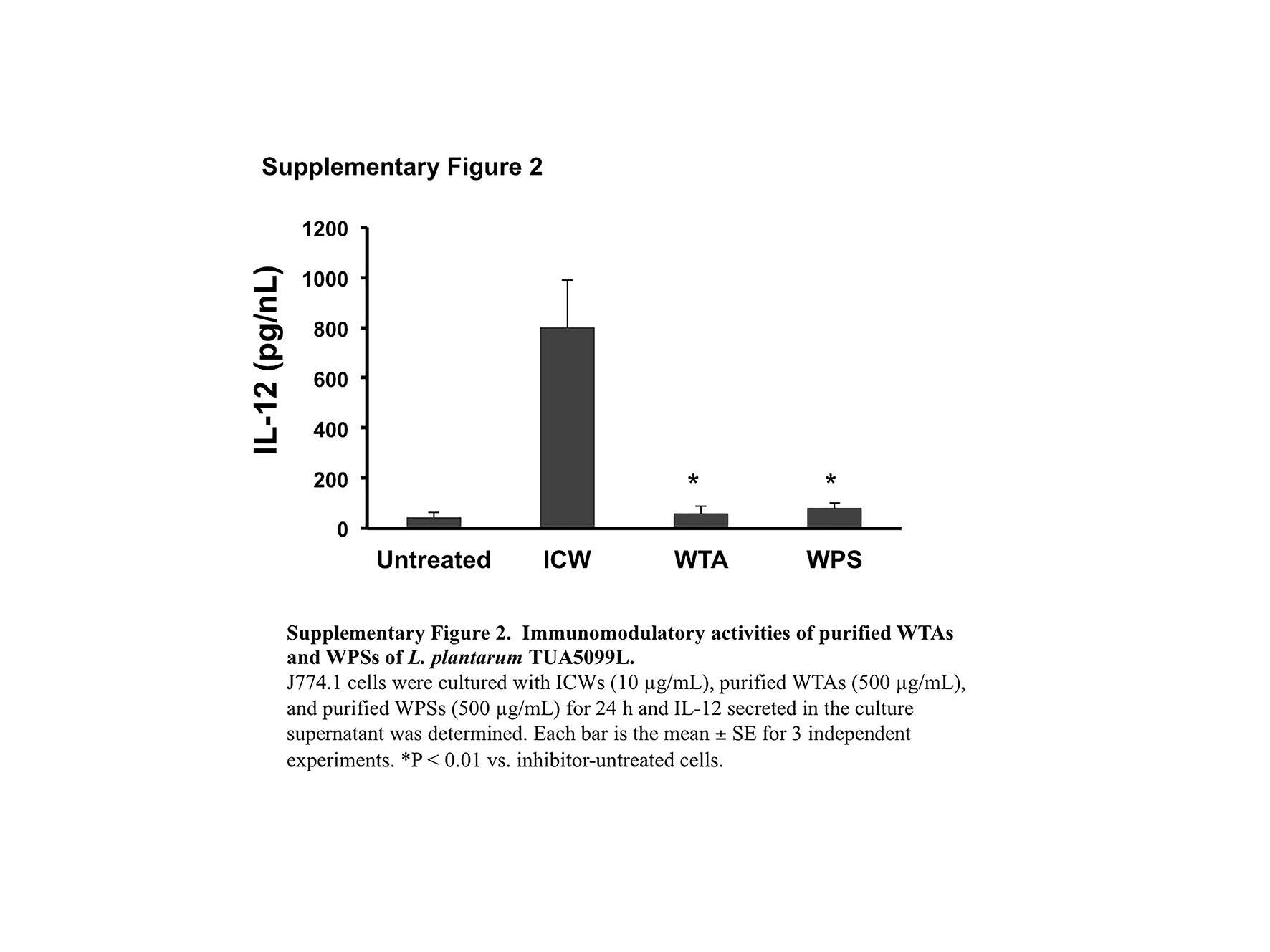

Supplement: Supplementary file 2 [file Image_2.JPEG]

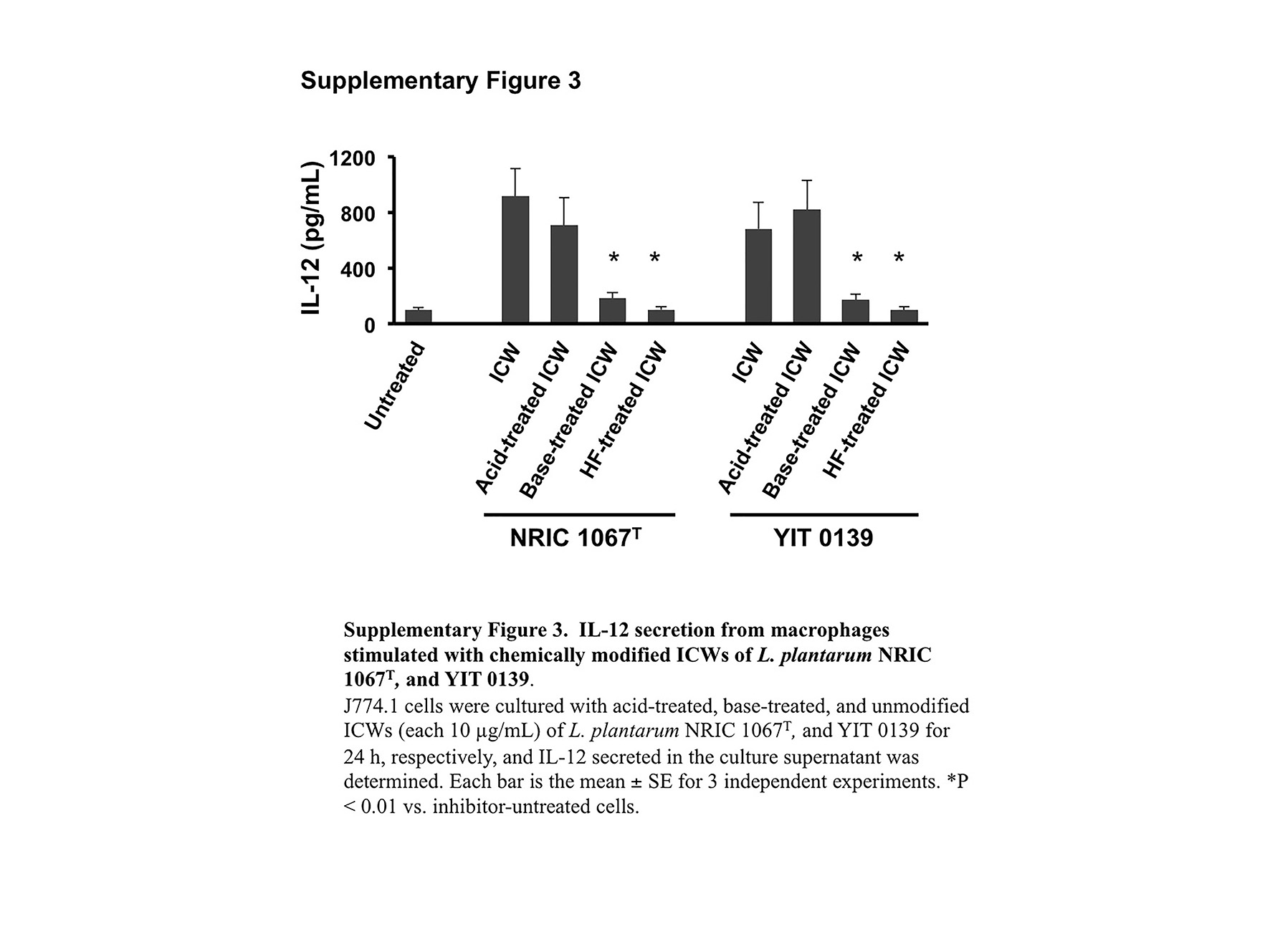

Supplement: Supplementary file 3 [file Image_3.JPEG]

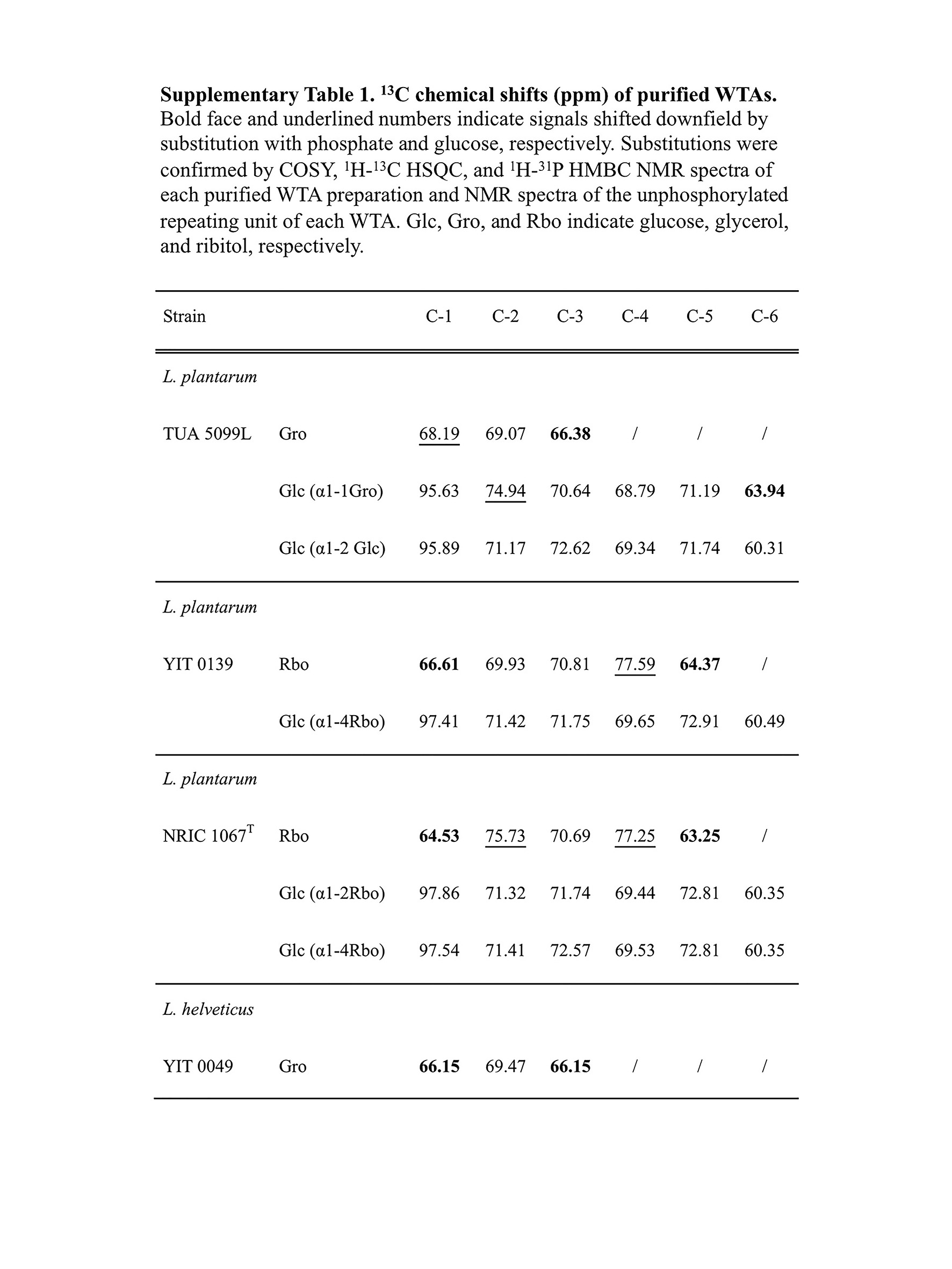

Supplement: Supplementary file 4 [file Image_4.JPEG]
